# Supplementary material for: Transduction of Salivary Gland Acinar Cells with a Novel AAV Vector 44.9
Source: Mol Ther Methods Clin Dev. 2020 Oct 14;19:459–66. doi: 10.1016/j.omtm.2020.10.006 (PMC7689275; doi:10.1016/j.omtm.2020.10.006)
Supplement: Document S1. Figures S1 and S2 [file mmc1.pdf]

OMTM, Volume *19*

Supplemental Information

Transduction of Salivary Gland Acinar Cells with a Novel AAV Vector 44.9

Giovanni Di Pasquale, Paola Perez Riveros, Muhibullah Tora, Tayyab Sheikh, Aran Son, Leyla Teos, Brigitte Grewe, William D. Swaim, Sandra Afione, Changyu Zheng, Shyh-Ing Jang, Akiko Shitara, Ilias Alevizos, Roberto Weigert, and John A. Chiorini

Figure S1. Acinar or ductal salivary glands cells have distinct membrane distribution of fluorescent reporter

Salivary glands from a mouse expressing the mTomato reporter (red) top right image, were fixed, processed for indirect immunofluorescence, labeled with the acinar marker NKCC1 (green), top left image, and phalloidin (blue), lower left image, then imaged by confocal microscopy, as described in the M&M section. In the merge image, lower right, NKCC1 positive acini (dashed white line) can be distinguished by their distribution of phalloidin from ducts (dashed yellow line) by their characteristic morphology and lumen structure. Yellow and white arrows in the insets highlight the basal membrane of the duct and acinar cell, respectively. Ductal cells exhibit a characteristic thicker distribution of the mTomato reporter membrane reporter due to the folding of the plasma membrane as also seen with phalloidin. Scale bar 10 µm.

Figure S2. Images of the salivary glands in mTomato/mGFP mice. The plasma membrane of salivary glands of CRE tomato/GFP mice has a strong red fluorescence that becomes green upon introduction of the CRE recombinase. Acini and ducts are structurally distinct as highlighted in the blue and yellow circles respectively (top image). Magnified images from Fig. 1 of positive acinar cells in mice transduced with the indicated AAV vector (lower images).

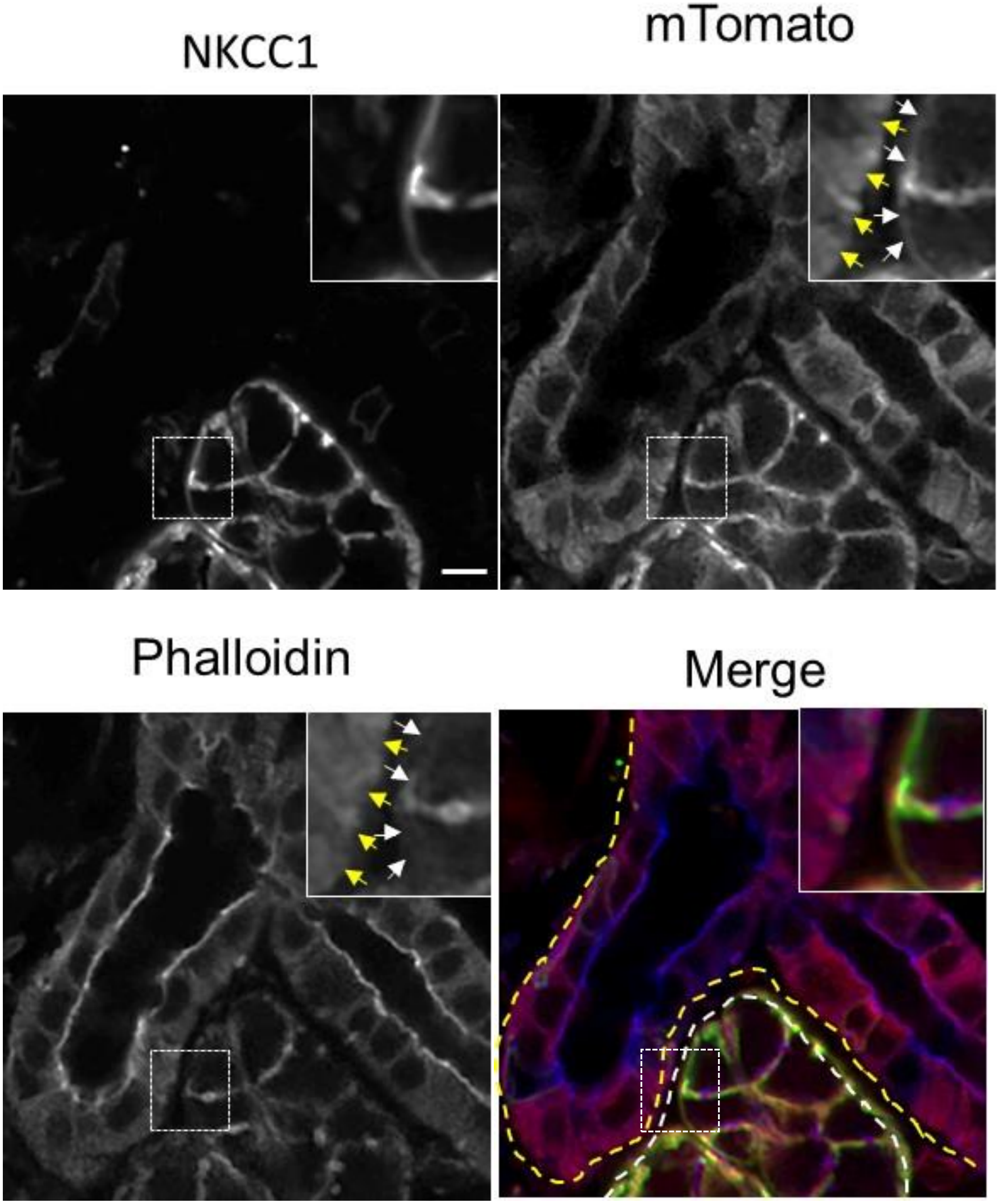

Supplemental Figure 1

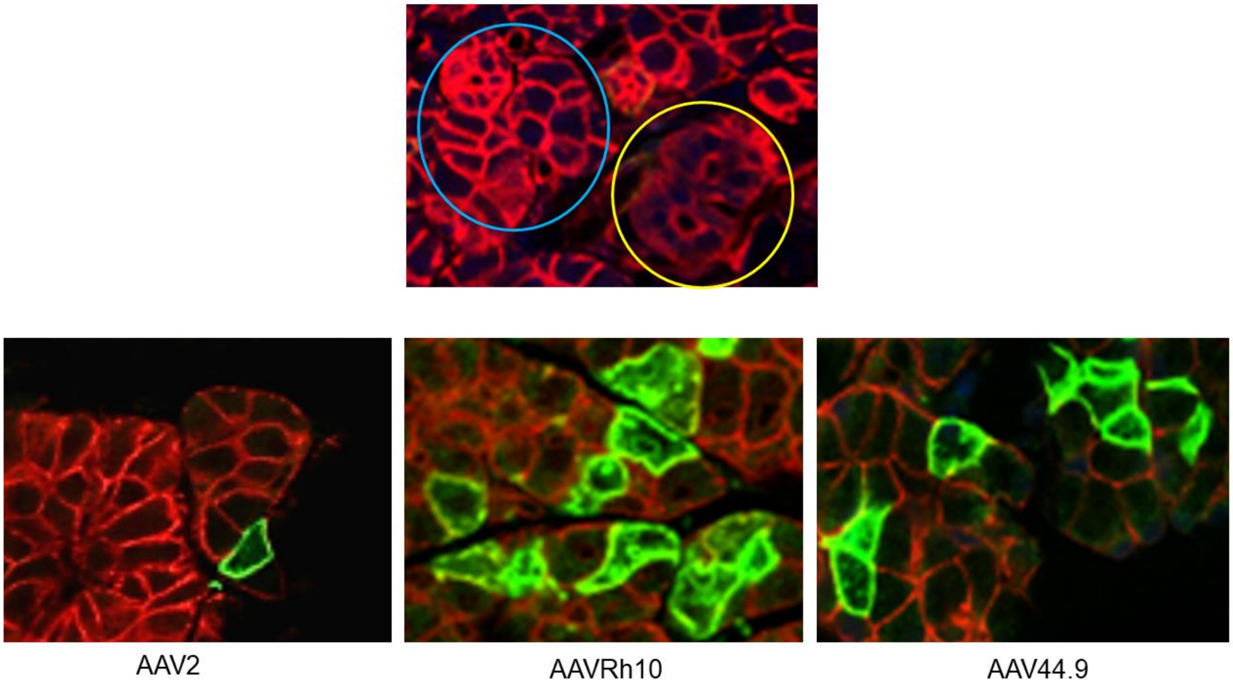

Supplemental Figure 2
