# Supplementary material for: Transduction of Salivary Gland Acinar Cells with a Novel AAV Vector 44.9
Source: Mol Ther Methods Clin Dev. 2020 Oct 14;19:459–66. doi: 10.1016/j.omtm.2020.10.006 (PMC7689275; doi:10.1016/j.omtm.2020.10.006)
Supplement: Document S2. Article plus Supplemental Information [file mmc2.pdf]

Molecular Therapy
Methods & Clinical Development
Original Article

Transduction of Salivary Gland Acinar Cells with a Novel AAV Vector 44.9

Giovanni Di Pasquale,[1] Paola Perez Riveros,[2] Muhibullah Tora,[3] Tayyab Sheikh,[1] Aran Son,[5] Leyla Teos,[4] Brigitte Grewe,[1] William D. Swaim,[2] Sandra Afione,[1] Changyu Zheng,[1] Shyh-Ing Jang,[2] Akiko Shitara,[3] Ilias Alevizos,[6] Roberto Weigert,[3] and John A. Chiorini[1]

[1]Adeno-Associated Virus Biology Section, National Institute of Dental and Craniofacial Research, National Institutes of Health, Bethesda, MD 20892, USA; [2]Salivary Gland Biology and Disorder Section, National Institute of Dental and Craniofacial Research, National Institutes of Health, Bethesda, MD 20892, USA; [3]Intracellular Membrane Trafficking Section, National Institute of Dental and Craniofacial Research, National Institutes of Health, Bethesda, MD 20892, USA; [4]Secretory Physiology Section, National Institute of Dental and Craniofacial Research, National Institutes of Health, Bethesda, MD 20892, USA; [5]Epithelial Signaling and Transport Section, National Institute of Dental and Craniofacial Research, National Institutes of Health, Bethesda, MD 20892, USA; [6]Sjögren's Syndrome and Salivary Gland Dysfunction Section, National Institute of Dental and Craniofacial Research, National Institutes of Health, Bethesda, MD 20892, USA

The loss of salivary gland function caused by radiation therapy of the head and neck or autoimmune disease such as Sjögren's syndrome is a serious condition that affects a patient's quality of life. Due to the combined exocrine and endocrine functions of the salivary gland, gene transfer to the salivary glands holds the potential for developing therapies for disorders of the salivary gland and the expression of therapeutic proteins via the exocrine pathway to the mouth, upper gastrointestinal tract, or endocrine pathway, systemically, into the blood. Recent clinical success with viral vector–mediated gene transfer for the treatment of irradiation-induced damage to the salivary glands has highlighted the need for the development of novel vectors with acinar cell tropism able to result in stable long-term transduction. Previous studies with adeno-associated virus (AAV) focused on the submandibular gland and reported mostly ductal cell transduction. In this study, we have screened AAV vectors for acinar cell tropism in the parotid gland utilizing membrane-tomato floxed membrane-GFP transgenic mice to screen CRE recombinase encoding AAV vectors of different clades to rapidly identify capsid isolates able to transduce salivary gland acinar cells. We determined that AAVRh10 and a novel isolate found as a contaminant of a laboratory stock of simian adenovirus SV15, AAV44.9, are both able to transduce parotid and sublingual acinar cells. Persistence and localization of transduction of these AAVs were tested using vectors encoding firefly luciferase, which was detected 6 months after vector administration. Most luciferase expression was localized to the salivary gland compared to that of distal organs. Transduction resulted in robust secretion of recombinant protein in both blood and saliva. Transduction was species specific, with AAVRh10 having stronger transduction activity in rats compared with AAV44.9 or AAV2 but weaker in human primary salivary gland cells. This work demonstrates efficient transduction of parotid acinar cells by AAV that resulted in secretion of recombinant protein in both serum and saliva.

INTRODUCTION

Collectively, salivary glands serve the critical exocrine role of producing saliva in the oral cavity. Saliva is essential for the lubrication, digestion, and protection of the oral cavity. The average individual produces approximately a liter of saliva per day, which will contain almost a gram of proteins. This represents an enormous secretory ability given the relatively small size of the salivary gland compared with other secretory tissue such as the liver. In addition, salivary glands also have the capacity to secrete proteins in an endocrine manner that traffics to the blood.

In mice, the submandibular gland is the largest salivary gland by volume whereas in humans the parotid gland is larger than the submandibular gland and is the gland responsible for most stimulated saliva production (reviewed in Maruyama et al.[1]). Because of the salivary gland's unique location and activity as both an endocrine and exocrine organ, gene transfer to this organ possesses the potential to treat genetic or acquired disease conditions. Furthermore, vectors can be easily delivered to the salivary gland by retroductal cannulation, allowing access to every cell within the gland.

The ultimate success of any gene therapy approach in the salivary gland is dependent on the efficient delivery of the therapeutic nucleic acid into the primary secretory cells (i.e., acinar cells). In the oral cavity, a number of approaches have been tried, ranging from injection of naked DNA, to engineered viruses and DNA lipid carrier complexes.[2] Vectors based on adeno-associated virus (AAV) have demonstrated long-term stable expression in a number of cell types *in vivo*. Previous reports of AAV vector transduction in the salivary gland have primarily focused on the submandibular gland of rodents and the parotid

Received 6 April 2020; accepted 9 October 2020;
https://doi.org/10.1016/j.omtm.2020.10.006.

Correspondence: John A. Chiorini, Adeno-Associated Virus Biology Section, National Institute of Dental and Craniofacial Research, National Institutes of Health, 10 Center Drive, Bld. 10, Room 1A21, Bethesda, MD 20892, USA.
E-mail: jchiorini@dir.nidcr.nih.gov

This is an open access article under the CC BY license (http://creativecommons.org/licenses/by/4.0/).

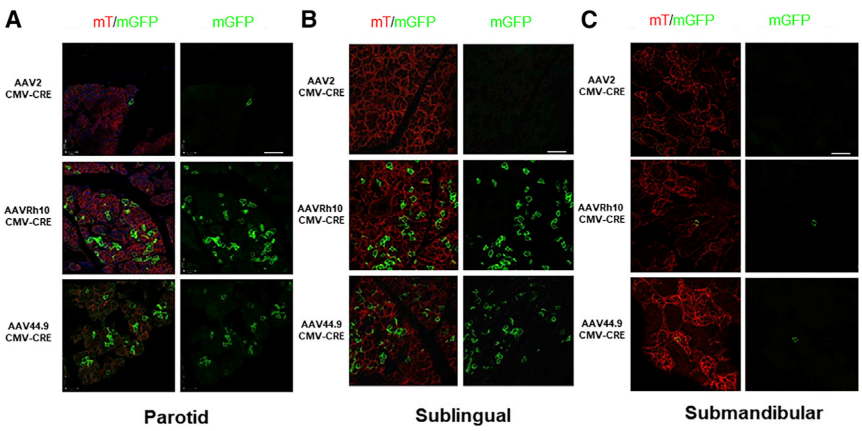

Figure 1. Transduction Efficiency and Specificity of Recombinant AAVs in Salivary Glands (SGs) of Tomato Mice

Each mouse received 2×10^{11} vector particles by cannulation of a single parotid SG. One month later tissue was collected and frozen sections of SGs were analyzed by confocal microscopy to examine transduction by looking for green positive cells (N = 3 per vector). (A) Parotid SG. (B) Sublingual SG. (C) Submandibular SG. The panel on the left is a merge of the Tomato and green fluorescence channels. The panel on the right is the green fluorescence channel only. Scale bars, 30 μm.

gland in non-human primates and minipigs.[3–5] Taken together, these studies demonstrate long-term expression primarily in ductal cells but little transduction in acinar cells. Since these reports, more than 100 AAV capsid sequences have been cloned from a variety of sources, and several serotypes have been reported to have transduction activity in salivary glands, including AAV2, AAV4, AAV5, BAAV, and AAV12.[4,6–8] Unfortunately, there is very little understanding of their host cell requirements for entry and transduction. What is known is that these viruses have evolved to utilize glycans, abundant on cell surfaces, as essential primary attachment points for entry. Many of these isolates can be grouped based on their glycan recognition; for example, AAV2, AAV3B, AAV6, and AAV13 all bind heparin sulfate proteoglycans while AAV1, AAV4, and AAV5 bind terminal sialic acid and AAV9 binds terminal galactose.[9–16] However, the binding specificity of many are unknown. Overall organization of all isolates is based on a clade system of phylogenetic relationships using computational approaches and not functional characterization, thereby relying on empirical observation in animal models to define tropism, which also maybe species specific.[17]

The goal of this study was to identify AAV vectors with parotid acinar cell tropism following retroductal cannulation. We observed that AAVRh10, a member of clade E, has enhanced transduction for acinar cells in the parotid gland. Interestingly, a novel isolate, AAV44.9, which falls between clade D and E and has homology to AAVRh8R, also had transduction activity for acinar cells in the parotid gland. Vectors delivered to the parotid gland also resulted in transduction of the sublingual but not the submandibular glands. The transduction was largely localized to these glands, persistent for at least 6 months, and resulted in increased protein expression in both blood and saliva.

RESULTS

AAV Transduces Salivary Gland Acinar Cells

To identify AAVs with increased transduction activity and persistence in salivary gland acinar cells, we screened AAVs from several clades. Transduction was compared using vectors encoding the CRE recombinase that were retroductally cannulated, as described in Materials and Methods, into a single parotid gland via Stensen's duct of an mT/mGFP transgenic mouse.[18,19] This strain ubiquitously expresses a membrane-targeted peptide fused with the tandem Tomato fluorescent protein. However, the expression of CRE recombinase by AAV vectors activates, in the transduced cells, the expression of a membrane-targeted peptide fused with the GFP (mGFP). Tissues section from the transduced animals were imaged by confocal fluorescence microscopy 1 month after cannulation. Nine capsid variants were tested in pilot experiments (AAV 2, 4, 5, 6, 8, 9, Rh10, BAAV, 44.9). Extensive cell transduction was observed with AAVRh10 and a new isolate AAV44.9 compared with AAV2 (Figure 1A). Acinar and ductal cells can be distinguished by their unique morphology, when visualized through the membrane-targeted peptide, as previously shown.[19–22] These differences were further confirmed by counterstaining the sections for (1) F-actin, as previously shown, and (2) the acinar cell protein NKCC1 (Figure S1). The observed AAVRh10 and AAV44.9 transduction could be clearly identified as predominately in acinar cells at high magnification (Figure S2). Cannulation of the parotid gland was via Stensen's duct located in the cheek, which is not connected to the submandibular and sublingual glands located on the floor of the mouth. Despite this anatomical separation, analysis of sublingual tissue sections showed extensive transduction of the sublingual gland but not the submandibular glands (Figures 1B and 1C). Quantification of acinar cell transduction by AAVRh10 and AAV44.9 showed that 10%–15% of parotid and sublingual cells were GFP-positive compared to less than 1% with AAV2 (Table 1).

Targeting of Salivary Gland Acinar Cells by AAVs Results in Long-Term and Localized Transduction

Activation of the Cre-Lox recombination in Tomato floxed GFP mice requires only transient or minimal expression of the Cre recombinase gene. Thus, GFP-positive cells observed in Figure 1 and quantified in Table 1 could be the result of transient transduction. Stability and biodistribution of expression were assessed by cannulating a single parotid gland of a mouse with AAV vectors encoding a cytomegalovirus (CMV)-luciferase reporter gene. Luciferase expression was monitored, in whole animals *in vivo*,[23] at 1 and 6 months after vector cannulation (Figures 2A–2C), as well as in isolated organs at 6 months (Figures 2D and 2E). At 1 month or 6 months after administration, all AAVs generated detectable luminescence in the neck region following

Table 1. Quantification of Positive Acinar Cells after Single Parotid Salivary Cannulation

	Parotid Glands (%)	Sublingual Glands (%)	Submandibular Glands (%)
AAV2	<1	<1	<1
AAVR10	9 ± 5	14 ± 5	<1
AAV44.9	10 ± 7	15 ± 5	<1

Percentage of positive (green) acinar cells were calculated by counting red and green cells. A total of 16 independent confocal microscope images were used for each vector.

luciferin substrate injection. However, the intensity was much higher in mice treated with AAVRh10 and AAV44.9 vectors compared with that of AAV2. AAVRh10 had an average increase in expression of 57- and 71-fold 1 or 6 months after cannulation, respectively, compared with AAV2, whereas AAV44.9 increased an average of 37- and 331-fold 1 or 6 months after cannulation, respectively, compared with AAV2 (Figure 2C). At 1 month after cannulation a weak luminescent signal was also observed in the abdomen area of AAVRh10- and AAV44.9-treated mice but not in AAV2-treated mice (Figure 2A). This signal represented less than 10% of that of the neck area detected at 1 month and was not detectable at the 6-month evaluation (Figure 2B).

AAV biodistribution was also measured by monitoring luciferase expression in isolated organs (Figure 2D). Luciferase expression was measured in salivary glands, stomach, spleen, lung, heart, kidney and liver. A more than 10-fold expression was detected in salivary glands of AAVRh10- and AAV44.9-treated mice compared with that of liver (Figures 2D and 2E). Lower levels of expression were detected in all other organs. AAVRh10 and AAV44.9 had a more than 150-fold expression in the salivary gland compared with that of AAV2 (Figure 2E).

Transduction of Salivary Gland Acinar Cells Results in Endocrine and Exocrine Secretion of the Recombinant *Gaussia* Luciferase Reporter

Acinar cells are highly specialized for secretion of both fluid and proteins. Although much of the secretory output is in the exocrine direction into the saliva via the regulated pathway, other research has demonstrated secretion via an endocrine pathway into the blood.[24,25] To assess the secretory potential of parotid acinar cells following transduction with AAV2, AAVRh10, or AAV44.9, one parotid gland in each mouse was cannulated with AAV vectors encoding *Gaussia* luciferase, which is reported to have a strong secretory signal peptide *in vitro* and *in vivo*.[26] Expression of *Gaussia* luciferase by each vector and secretion into the serum and saliva were tested at 8 months after cannulation and quantified by comparing luminescence values generated from purified recombinant *Gaussia* luciferase (see Materials and Methods).

Although relatively low compared to stimulated saliva, secretion of recombinant protein could be detected in basal unstimulated saliva secretion (collected as an oral wash) as well as in pilocarpine/isoproterenol-stimulated saliva secretion (Figure 3). Oral wash (see Materials and Methods) yielded 13.3 ± 3.3 and 10 ± 3.3 pg/mL of luciferase protein for AAVRh10- or AAV44.9-treated mice, respectively, whereas luciferase was undetectable in the saliva of mice treated with AAV2 (Figure 3A). In contrast, stimulated secretion yielded 39,000 ± 2,000 and 35,000 ± 1,000 pg/mL of luciferase in AAVRh10- or AAV44.9-treated mice, respectively (Figure 3A). Stimulated parotid luciferase secretion was below the level of detection in AAV2-treated mice (Figure 3A).

Serum of mice cannulated with AAVRh10 or AAV44.9 contained more than 338 ± 25 and 328 ± 21 pg/mL, respectively, of *Gaussia* luciferase, whereas serum from AAV2-treated mice was similar to control untreated mice (Figure 3B).

Taken together, these data show that transduction with AAVRh10 or AAV44.9 can result in sustained secretion in saliva and serum. Recombinant protein secretion was also observed, in unstimulated saliva, whereas stimulation led to at least a 3,000-fold increase.

AAV Salivary Gland Acinar Cells Transduction Is Species Specific

In order to verify whether AAV acinar cell transduction is species-independent, we tested AAV transduction in rat parotid salivary glands and AAV transduction in human primary acinar cells organocultures derived from human minor salivary glands biopsies (Figure 4). In contrast to mice, cannulation and infusion of rat parotid glands with AAV vectors encoding firefly luciferase resulted in expression of only AAVRh10, but not with AAV2 or AAV44.9 (Figures 4A and 4B). AAVRh10 cannulation resulted in secretion of recombinant protein in both the saliva and serum (data not shown).

The development of primary human salivary gland cultures has provided useful models for studying acinar cell function.[27] Recent work has demonstrated that primary human minor salivary gland (phSG) cell cultures achieve an acinar-like phenotype and exhibit progenitor cell markers with acinar-specific markers. In addition, these cells form a monolayer with high transepithelial electrical resistance.[27]

phSG cells were seeded in Transwell membrane dishes and allowed to establish an acinar cell phenotype prior to incubation with single-stranded (Figures 4C and 4D) or self-complementary AAV2, AAVRh10, or AAV44.9 vectors encoding a CMV-GFP reporter, respectively (Figure 4D). Transduction with AAV2 could be observed as soon as 24 h after incubation (data not shown). Five days after incubation with vector, self-complementary AAV2 and AAV44.9 were detected in 95% ± 5% and 35% ± 10% of the cells, respectively, whereas no transduction was detected with AAVRh10 (Figure 4D). Single-stranded AAV2 also showed the highest transduction activity of phSG cells compared with AAV44.9 and AAVRh10 (Figures 4C and 4D). Taken together, these data suggest that salivary gland acinar cells of different species are differentially permissive to AAV isolates.

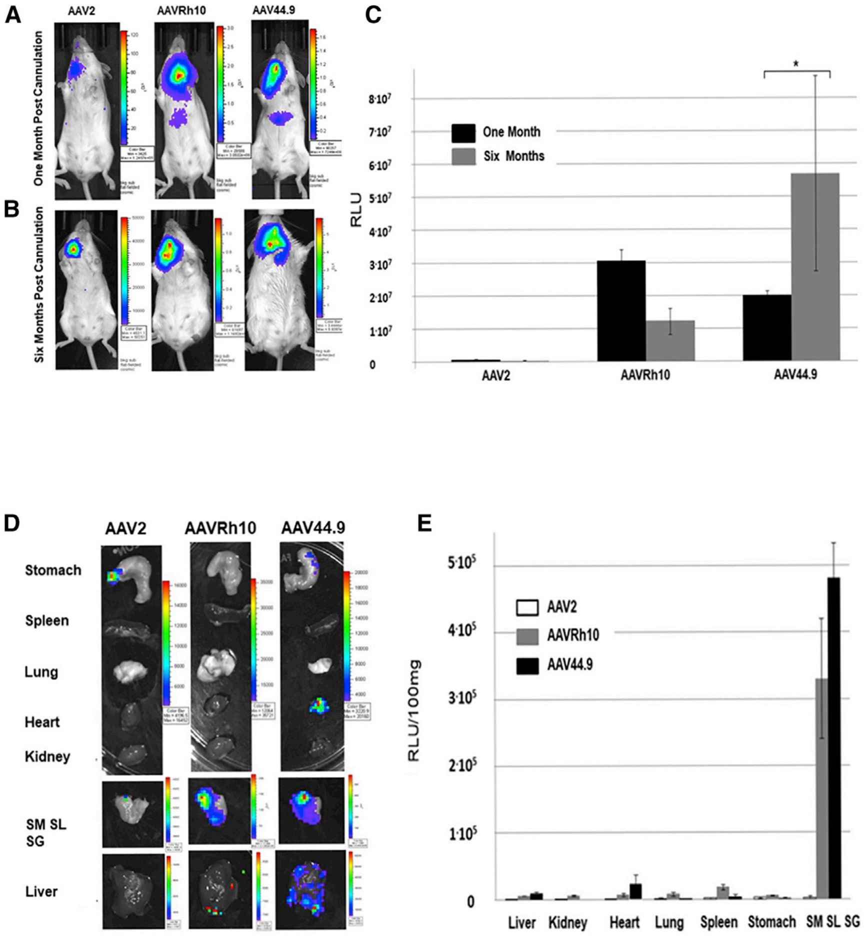

Figure 2. *In Vivo* Long-Term Transduction

Each mouse was treated with 2×10^{10} vector particles to a single parotid SG. (A and B) Luciferase expression was monitored in live animals and quantified in SGs and liver 1 month (A) or 6 months (B) after cannulation by a Xenogen IVIS. (C) Quantification histogram chart of experiments shown in (A) and (B) (N = 4). $^*p < 0.05$. Each of the organs was individually analyzed for luciferase expression. (D) Representative organs from mice cannulated with 2×10^{10} AAVs in a volume of 50 μL analyzed by a Xenogen IVIS. (E) Luminescence quantification histogram of experiments shown in (D) (N = 4).

DISCUSSION

The development of salivary gland-targeted gene therapy could be useful for treating both diseases of the salivary gland and systemic diseases because of the natural exocrine and endocrine secretory activity of the gland. In this study, we have shown that several new clades of AAV are able to transduce acinar cells. Gene transfer with adenovirus has been extensively tested in the salivary gland because of its robust expression of recombinant proteins and broad cell tropism. However, adenovirus transduction is reported to be transient in animal models and can initiate a strong immune reaction to the transduced cells, limiting its clinical utility. The use of AAV in salivary gland gene transfer application offers an immunological advantage over adenovirus, although the ductal cell tropism reported in the submandibular glands might limit its application. Discovery of new clades have improved gene transfer in organs like liver, heart, CNS, and muscle. Some of these new isolates have the capability, when administrated systemically, to transcytose across the blood-brain barrier and are currently in clinical trials for neurodegenerative diseases.[28–30]

In this study, retroductal cannulation and infusion of either AAVRh10 or AAV44.9 vector resulted in transduction of parotid acinar cells; surprisingly, transduction was not limited to the cannulated parotid gland but also transduced the acinar cells of the sublingual gland. We did not observe transduction in the submandibular gland following this route of delivery (Figures 1A–1C) or transduction in the contralateral parotid gland. The mechanism of transduction of these two major salivary glands is not clear since they have separate secretory ducts, and thus it is unclear how the vector could reach the sublingual gland. An alternative route of delivery may be via the lymphatic system, which connects these glands.[31] Following parotid delivery, lateral lymph nodes associated with these salivary glands may offer limited local circulation of AAV between these glands.

The observed AAV transduction persisted for at least 6 months. Although the vast majority of the luciferase signal was detected in the cannulated salivary gland (>20-fold), some transduction was detected in the liver or heart. Minor expression in distal organs was not surprising considering the capability of some isolates to spread.[28] Future studies utilizing acinar cell-specific promoters could limit some of this off-target transduction. Salivary gland-targeted gene therapy is currently in clinical trial for patients with radiation-induced salivary hypofunction as a result of treatment for oral cancer (ClinicalTrials.gov: NCT02446249). Several studies have suggested that salivary gland-targeted gene therapy may represent an effective method of treating the inflammation and loss of gland function associated with the autoimmune disease Sjögren's syndrome.[19,32–37] Furthermore, due to the natural secretory activity of the salivary gland and ability to traffic recombinant proteins to the saliva or serum, transduction of the salivary gland may offer a novel route of delivery for the treatment of systemic disease.[25] As a proof of concept to test the secretory capacity of the salivary gland, we have used *Gaussia* luciferase, which has a potent signal peptide that is reported to drive the secretion of 90% of the protein expressed both *in vitro* and *in vivo*.[26] Cannulation of the salivary gland with AAVRh10 or AAV44.9 encoding the *Gaussia* luciferase gene resulted in secretion of ∼40 ng/mL or ∼350 pg/mL in saliva and serum, respectively, for at least 8 months after administration. *Gaussia* luciferase was recovered and quantified in both the unstimulated and stimulated saliva secretions, confirming that expression

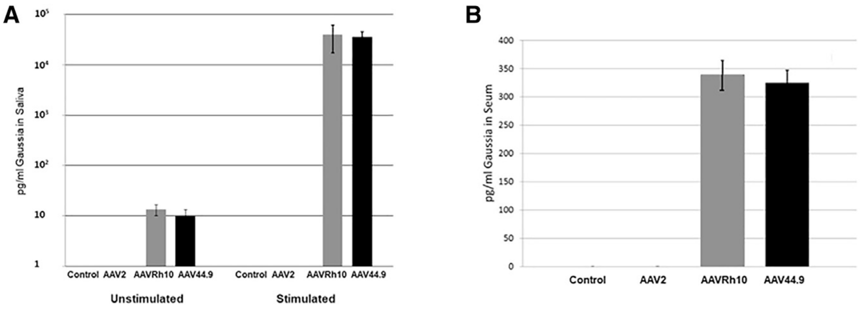

Figure 3. Exocrine and Endocrine Release of Recombinant Protein after Parotid Cannulation of AAV Vectors

(A and B) Eight months after AAVs administration, luciferase secretion was quantified by a *Gaussia* luciferase assay in (A) unstimulated saliva or stimulated saliva or in (B) mouse serum (N = 4). In (A), the oral cavity of unstimulated treated mice was rinsed with total volume of 150 μL of PBS and then assayed in a *Gaussia* luciferase assay. Stimulated secretion of *Gaussia* luciferase was quantified in saliva by first injecting pilocarpine and isoproterenol and then collecting the pooled saliva from the floor of the mouth with a pipette.

was from the salivary gland epithelia and that a basal level of secretion is possible. This level of protein secretion is in the physiologic range of hormones such as glucagon-like peptide-1 and parathyroid hormone.

Currently, it is not clear what is the signal peptide for endocrine secretion in the salivary gland. Recent work suggests that it may differ between secreted proteins in the parotid and submandibular glands.[38]

To understand whether the differences in acinar cells transduction activity observed in the parotid gland were observed across species, salivary gland gene transfer was also studied in rats and human primary salivary glands cells. Interestingly, only AAVRh10 showed strong transduction activity in rats but little transduction in human cells. A difference in salivary gland transduction between mice and rats has been previously observed.[6] Alternatively, AAV2, which had lower transduction in both mouse and rat acinar cells, was by far the most efficient in the human cells. It is not clear whether this is the result of *in vitro* versus *in vivo* conditions or the result of immersion of the cells in vector versus apical delivery via cannulation. Taken together, the data suggest that salivary gland cells are differentially permissive for AAVs and there are species-specific differences to transduction. Similar observations have been reported in other organs such as the lung.[39]

For human gene therapy applications targeting acinar cells, tropism studies in other non-rodent animal models such as minipigs or non-human primates are necessary.

Understanding the biological basis of these differences in virus host cell interactions would be important in translating preclinical work into successful clinical outcomes.

Identification of new isolates with improved gene transfer activity and a better understanding of species-specific AAV transduction activity should lead to improved gene transfer vectors. Further improvements in gland transduction activity are likely by directed evolution of the capsids of these isolates to improve their transduction and limit spread to other organs.

MATERIALS AND METHODS

Animal Models

Tomato floxed GFP mice and B6.129(Cg)-*Gt(ROSA)26Sor*$^{tm4(ACTB-tdTomato,-EGFP)Luo}$/J, BALB/c, and C57BL/6 mice were obtained from The Jackson Laboratory. Wistar rats were also obtained from The Jackson Laboratory.

Animals were housed in a pathogen-free facility. All procedures involving live animals were performed in an accredited vivarium according to institutional guidelines and standard operating procedures and were in compliance with the NIH *Guide for the Care and Use of Laboratory Animals*.

Mouse and Rat Parotid Salivary Semi-flexible Cannulas

Rodent salivary gland cannulation has been described previously.[2]

In this study, we modified the previously reported thin polyethylene tube (PE 10) cannula used for mice with a semi-flexible one. The cannula was constructed by inserting a MicroFil custom 35G nonmetallic needle (1.5 inches long) (World Precision Instruments [WPI], #CMF35G) into polyethylene tubing (Strategic Applications, #PE-5-100). The joint was then sealed using a paper strip treated with cyanoacrylate adhesive (superglue). The other end of the cannula was connected to a 31G insulin syringe (Becton Dickinson). Cannulas were tested before use for reliable loading and delivery of infusate without leaking.

For cannulation of rats, an 8-mm 31G needle with the needle removed from an insulin syringe bluntly cut with a scissor was connected to polyethylene tubing from the beveled side. The other end of the cannula was connected to an insulin syringe.

Parotid Salivary Gland Cannulation

Animals were anesthetized by intramuscular injection with 5 mL/kg of a cocktail containing ketamine (43 mg/kg) and xylazine (8.5 mg/kg), using additional anesthetic as needed. Saline was applied to the eyes to prevent dryness. In order to easily access the Stensen's duct located in the lateral surface of the mouth, mice or rats were maintained in a reclined position, and the mouth was kept open by restraining the cheeks with two suture hooks. Using a surgical microscope (Leica MZ7s), the appropriate cannula was inserted into the parotid duct orifice using forceps and sealed in place with superglue on the surrounding buccal fat pad. After 10 min, the dose of rAAV vector was infused into the gland via the cannula connected to the insulin syringe.

Ineffective cannulation was monitored by checking for sample leakage around the ductal opening following infusion or when the

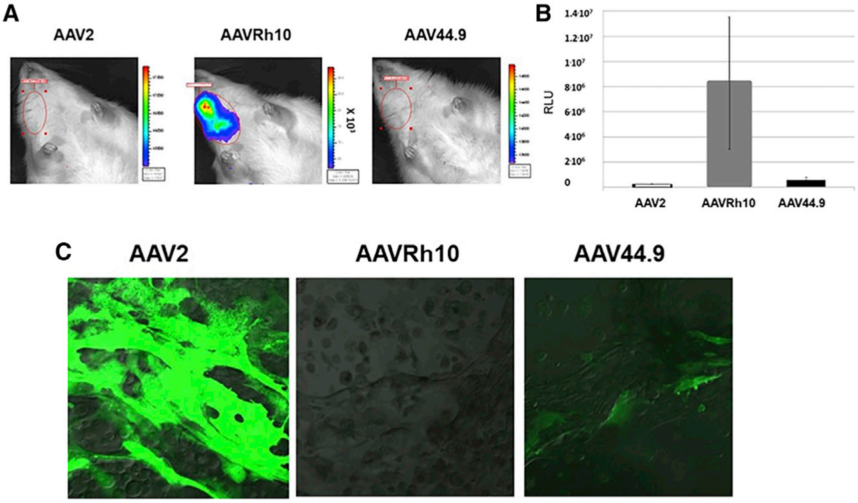

D

	AAV2	AAVRh10	AAV44.9
Single Stranded	85 +/- 10%	<1%	10 +/- 5%
Self Complementary	95 +/- 5%	<1%	35 +/-10 %

Figure 4. AAV SG Acinar Cell Transduction in Rats and Primary Human Organoculture

Rats were treated by single parotid SG cannulation of 1.5 × 10[11] AAVs encoding firefly luciferase. (A and B) Luciferase expression was live monitored (A) and quantified (B) in SGs, 1 month later, by a Xenogen IVIS (N = 3). (C and D) Transduction efficiency of AAVs in primary cultures from human SGs. Cells were incubated with 5 × 10[4] viral particles per cell of self-complementary or single-stranded AAVs encoding the GFP reporter gene. (C) Five days after incubation with single-stranded vectors, cells were imaged by fluorescence microscopy. (D) Quantification of self-complementary or single-stranded AAVs (N = 3).

buccal fat pad around the orifice started to inflate. In such rare cases the animals were excluded from the experiment cohort.

Following cannulation, the device was held in place for 10 min, then removed. During recovery, mice or rats were placed in an oxygen chamber until ambulatory.

Confocal Immunofluorescent Imaging Microscopy

The salivary glands of mTomato/mGFP mice were fixed in 4% formaldehyde overnight at 4°C and then frozen in liquid nitrogen and stored at −80°C. Cryosections were cut using a Leica CM3050S cryostat, after which samples were mounted in Fluoromount G on a glass slides and covered with a #1.5 coverslip. Immunostaining was performed as follows. Samples were incubated (1) in 10% fetal bovine serum (FBS) and (2) 0.02% saponin in FBS (blocking solution) for 30–45 min at room temperature with primary goat antibodies (NKCC1, 1:00; N-16, Santa Cruz) at 4°C overnight; (3) with secondary antibodies anti- goat Alexa Fluor 488 in blocking solution at 4°C for 30 min; and (4) if needed, with phalloidin-iFluor 405 (Abcam) for 30–60 min at room temperature. Finally, samples were mounted in Fluoromount G on a glass slide and covered with a #1.5 coverslip. Confocal images were acquired using a FluoView 1000 (Olympus).

Live Body Imaging of Luciferase Expression

Mice were anesthetized in an isoflurane vaporizing chamber and then injected with 200 μL of luciferase substrate D-luciferin potassium salt (GoldBio) dissolved in 40 mg/mL PBS intraperitoneally. Mice were then transferred to a Lumina *in vivo* Imaging System (IVIS) dark chamber equipped with an anesthetic vaporizer and imaged. Luciferase activity was quantified using Living Image software, version 2.60.1 (Caliper Life Sciences, Alameda, CA, USA).

For quantification, each image is shown with its own luminescence scale bar.

Luciferase Expression Quantification by Imaging

Five minutes after treatment with anesthesia, mice were injected with luciferase substrate and the whole body was imaged. Following completion of the whole-body imaging, animals were euthanized in a CO_2 chamber with each organ isolated and imaged as described above. Luciferase activity was quantified using Living Image software.

Production and Purification of Recombinant AAVs

Different isolates of recombinant AAV particles were produced using a four-plasmid procedure as previously described.[18] Human embryonic kidney 293T cells were obtained from the American Type Culture Collection (ATCC, Manassas, VA, USA). 293T cells were grown at 37°C under a 5% CO_2 humidified atmosphere in Dulbecco's modified Eagle's medium supplemented with 10% FBS, 2 mM L-glutamine, 100 U of penicillin/mL, and 0.1 mg of streptomycin/mL. Briefly, 293T cells were transfected by calcium phosphate with four plasmids as follows: an adenovirus helper plasmid (pAd12) containing VA RNA and coding the E2 and E4 proteins; two AAV helper plasmids containing either the AAV rep or the isolate-specific capsid gene; and a vector plasmid including the AAV2 inverted terminal repeats flanking the appropriate reporter gene expression cassette. The cells were harvested 48 h post-transfection and a crude viral lysate was obtained after one freeze-thaw cycle. The lysate was treated with 0.5% deoxycholic acid (DOC) and 100 U/mL DNase (Benzonase) for 30 min at 37°C. The vector particles present in the clarified lysate (obtained by further low-speed centrifugation) were further purified by CsCl gradient ultracentrifugation, and the vector titer was determined by quantitative real-time PCR (Applied Biosystems, Foster City, CA, USA). The vector doses were dialyzed against 0.9% NaCl using Slide-A-Lyzer 10K cassettes (Thermo Fisher Scientific). Vector was concentrated using a centrifugal filter unit (Amicon Ultra).

Blood and Saliva Collection

Blood samples were withdrawn from mice or rats under isoflurane anesthesia by retro-orbital bleeding and collected in a BD Microtainer.

Serum was separated by centrifugation and immediately frozen and stored on dry ice or in a −80°C freezer. Stimulated saliva was induced in mice under isoflurane anesthesia by administrating a cocktail of freshly prepared pilocarpine (0.5 mg/kg) and isoproterenol (0.1 mg/kg) in the nape of the neck. Saliva was collected during 10 min using a 200-μL capillary pipette placed from the oral cavity into chilled 1.5-mL microcentrifuge tubes and stored at −20°C or below. More than 100 μL of a saliva sample was collected from each mouse or 300 μL from each rat. An oral wash in unstimulated mice was performed by rinsing the oral cavities with 150 μL of PBS and immediately freezing the collected samples for *Gaussia* luciferase assays.

Gaussia Luciferase Assay in Saliva or Serum

Gaussia luciferase activity was measured by combining 10 μL of saliva diluted in 90 μL of PBS and transferred, in duplicate, into white CulturPlate-96 wells (PerkinElmer). 100 μL of *Gaussia* buffer substrate (Pierce *Gaussia* glow assay kit) was added to the sample and immediately analyzed and quantified in a luminometer (PerkinElmer Victor X2). Similarly, 30 μL of serum was diluted in 70 μL of PBS and then treated as for the saliva. In oral wash experiments, 100 μL of sample was used together with 100 μL of substrate.

Relative light unit (RLU) values obtained from saliva and serum sample analyses were converted to a protein concentration by generating a standard curve using RLU values obtained by serial dilution of known purified recombinant *Gaussia* luciferase (NanoLight Tech).

Primary Human Salivary Gland Cells

phSG cells were obtained through serial passage of cells from biopsy tissue expanded on collagen-coated plates (BioCoat, Becton Dickinson) as previously described.[27] Briefly, phSG cells were maintained in complete keratinocyte growth medium (KGM) (Lonza) supplemented with bovine pituitary extracts (BPEs), recombinant human epidermal growth factor (rhEGF), insulin (INS), hydrocortisone (HC), gentamicin, epinephrine, and transferrin, and the calcium concentration was adjusted to 0.05 mM with $CaCl_2$ solution. Differentiation was induced in high Ca^{2+}, 1.2 mM KGM for 3 days prior to transduction with AAV vectors.[27] Transduction was monitored by GFP expression at the indicated time.

Statistical Analysis

Data were analyzed using Excel software. Continuous variables with normal distributions are shown as mean ± standard deviation (SD).

SUPPLEMENTAL INFORMATION

Supplemental Information can be found online at https://doi.org/10.1016/j.omtm.2020.10.006.

AUTHOR CONTRIBUTIONS

G.D.P. conceived, coordinated, and performed experiments and wrote the manuscript. J.A.C. conceived and wrote the manuscript and secured funding. R.W. conceived and wrote the manuscript. I.A. conceived experiments. P.P.R., M.T., and A.S., provided expertise and performed experiments. T.S., A.S., L.T., B.G., W.D.S., S.A., C.Z., and S.-I.J. performed experiments.

CONFLICTS OF INTEREST

The authors declare no competing interests.

ACKNOWLEDGMENTS

The authors would like to thank members of the AAV Biology section for assistance with this work. This work was supported by an NIDCR intramural grant to J.A.C. (1Z1ADE000695) and the Veterinary Resources Core (ZIC DE000740-05).

REFERENCES

1. Maruyama, C.L., Monroe, M., Hunt, J., Buchmann, L., and Baker, O.J. (2019). Comparing human and mouse salivary glands: a practice guide for salivary researchers. Oral Dis. *25*, 403–415.
2. Baum, B.J., Alevizos, I., Chiorini, J.A., Cotrim, A.P., and Zheng, C. (2015). Advances in salivary gland gene therapy—oral and systemic implications. Expert Opin. Biol. Ther. *15*, 1443–1454.
3. Hai, B., Yan, X., Voutetakis, A., Zheng, C., Cotrim, A.P., Shan, Z., Ding, G., Zhang, C., Xu, J., Goldsmith, C.M., et al. (2009). Long-term transduction of miniature pig parotid glands using serotype 2 adeno-associated viral vectors. J. Gene Med. *11*, 506–514.
4. Katano, H., Kok, M.R., Cotrim, A.P., Yamano, S., Schmidt, M., Afione, S., Baum, B.J., and Chiorini, J.A. (2006). Enhanced transduction of mouse salivary glands with AAV5-based vectors. Gene Ther. *13*, 594–601.
5. Voutetakis, A., Zheng, C., Mineshiba, F., Cotrim, A.P., Goldsmith, C.M., Schmidt, M., Afione, S., Roescher, N., Metzger, M., Eckhaus, M.A., et al. (2007). Adeno-associated virus serotype 2-mediated gene transfer to the parotid glands of nonhuman primates. Hum. Gene Ther. *18*, 142–150.
6. Braddon, V.R., Chiorini, J.A., Wang, S., Kotin, R.M., and Baum, B.J. (1998). Adenoassociated virus-mediated transfer of a functional water channel into salivary epithelial cells in vitro and in vivo. Hum. Gene Ther. *9*, 2777–2785.
7. Schmidt, M., Katano, H., Bossis, I., and Chiorini, J.A. (2004). Cloning and characterization of a bovine adeno-associated virus. J. Virol. *78*, 6509–6516.
8. Schmidt, M., Voutetakis, A., Afione, S., Zheng, C., Mandikian, D., and Chiorini, J.A. (2008). Adeno-associated virus type 12 (AAV12): a novel AAV serotype with sialic acid- and heparan sulfate proteoglycan-independent transduction activity. J. Virol. *82*, 1399–1406.
9. Bell, C.L., Gurda, B.L., Van Vliet, K., Agbandje-McKenna, M., and Wilson, J.M. (2012). Identification of the galactose binding domain of the adeno-associated virus serotype 9 capsid. J. Virol. *86*, 7326–7333.
10. Shen, S., Bryant, K.D., Brown, S.M., Randell, S.H., and Asokan, A. (2011). Terminal *N*-linked galactose is the primary receptor for adeno-associated virus 9. J. Biol. Chem. *286*, 13532–13540.
11. Govindasamy, L., Padron, E., McKenna, R., Muzyczka, N., Kaludov, N., Chiorini, J.A., and Agbandje-McKenna, M. (2006). Structurally mapping the diverse phenotype of adeno-associated virus serotype 4. J. Virol. *80*, 11556–11570.
12. Kaludov, N., Brown, K.E., Walters, R.W., Zabner, J., and Chiorini, J.A. (2001). Adeno-associated virus serotype 4 (AAV4) and AAV5 both require sialic acid binding for hemagglutination and efficient transduction but differ in sialic acid linkage specificity. J. Virol. *75*, 6884–6893.
13. Lerch, T.F., Xie, Q., and Chapman, M.S. (2010). The structure of adeno-associated virus serotype 3B (AAV-3B): insights into receptor binding and immune evasion. Virology *403*, 26–36.
14. Ng, R., Govindasamy, L., Gurda, B.L., McKenna, R., Kozyreva, O.G., Samulski, R.J., Parent, K.N., Baker, T.S., and Agbandje-McKenna, M. (2010). Structural characterization of the dual glycan binding adeno-associated virus serotype 6. J. Virol. *84*, 12945–12957.

15. Walters, R.W., Yi, S.M., Keshavjee, S., Brown, K.E., Welsh, M.J., Chiorini, J.A., and Zabner, J. (2001). Binding of adeno-associated virus type 5 to 2,3-linked sialic acid is required for gene transfer. J. Biol. Chem. *276*, 20610–20616.

16. Wu, Z., Miller, E., Agbandje-McKenna, M., and Samulski, R.J. (2006). α2,3 and α2,6 N-linked sialic acids facilitate efficient binding and transduction by adeno-associated virus types 1 and 6. J. Virol. *80*, 9093–9103.

17. Gao, G., Vandenberghe, L.H., Alvira, M.R., Lu, Y., Calcedo, R., Zhou, X., and Wilson, J.M. (2004). Clades of adeno-associated viruses are widely disseminated in human tissues. J. Virol. *78*, 6381–6388.

18. Muzumdar, M.D., Tasic, B., Miyamichi, K., Li, L., and Luo, L. (2007). A global double-fluorescent Cre reporter mouse. Genesis *45*, 593–605.

19. Milberg, O., Shitara, A., Ebrahim, S., Masedunskas, A., Tora, M., Tran, D.T., Chen, Y., Conti, M.A., Adelstein, R.S., Ten Hagen, K.G., and Weigert, R. (2017). Concerted actions of distinct nonmuscle myosin II isoforms drive intracellular membrane remodeling in live animals. J. Cell Biol. *216*, 1925–1936.

20. Shitaraa, A., Maleca, L., Ebrahima, S., Chena, D., Bleckc, C., Hoffmand, M.P., and Weigerta, R. (2019). Cdc42 negatively regulates endocytosis during apical membrane maintenance in live animals. Mol. Biol. Cell *30*, 324–332.

21. Masedunskasa, A., Sramkovaa, M., Parentea, L., Salesc, K.U., Amornphimoltham, P., Buggec, T.H., and Weigert, R. (2011). Role for the actomyosin complex in regulated exocytosis revealed by intravital microscopy. Proc. Natl. Acad. Sci. USA *108*, 13552–13557.

22. Sramkova, M., Masedunskas, A., Parente, L., Molinolo, A., and Weigert, R. (2009). Expression of plasmid DNA in the salivary gland epithelium: novel approaches to study dynamic cellular processes in live animals. Am. J. Physiol. Cell Physiol. *297*, C1347–C1357.

23. Di Pasquale, G., Kaludov, N., Agbandje-McKenna, M., and Chiorini, J.A. (2010). BAAV transcytosis requires an interaction with β-1-4 linked- glucosamine and gp96. PLoS ONE *5*, e9336.

24. Di Pasquale, G., et al. (2012). Sustained exendin-4 secretion through gene therapy targeting salivary glands in secretion through gene therapy targeting salivary glands in two different rodent models of obesity/type 2 diabetes. PLoS One *7*, e40074.

25. Yamano, S., Huang, L.Y., Ding, C., Chiorini, J.A., Goldsmith, C.M., Wellner, R.B., Golding, B., Kotin, R.M., Scott, D.E., and Baum, B.J. (2002). Recombinant adeno-associated virus serotype 2 vectors mediate stable interleukin 10 secretion from salivary glands into the bloodstream. Hum. Gene Ther. *13*, 287–298.

26. Tannous, B.A., Kim, D.E., Fernandez, J.L., Weissleder, R., and Breakefield, X.O. (2005). Codon-optimized *Gaussia* luciferase cDNA for mammalian gene expression in culture and in vivo. Mol. Ther. *11*, 435–443.

27. Jang, S.I., Ong, H.L., Gallo, A., Liu, X., Illei, G., and Alevizos, I. (2015). Establishment of functional acinar-like cultures from human salivary glands. J. Dent. Res. *94*, 304–311.

28. Di Pasquale, G., and Chiorini, J.A. (2006). AAV transcytosis through barrier epithelia and endothelium. Mol. Ther. *13*, 506–516.

29. Duque, S., Joussemet, B., Riviere, C., Marais, T., Dubreil, L., Douar, A.-M., Fyfe, J., Moullier, P., Colle, M.-A., and Barkats, M. (2009). Intravenous administration of self-complementary AAV9 enables transgene delivery to adult motor neurons. Mol. Ther. *17*, 1187–1196.

30. Foust, K.D., Nurre, E., Montgomery, C.L., Hernandez, A., Chan, C.M., and Kaspar, B.K. (2009). Intravascular AAV9 preferentially targets neonatal neurons and adult astrocytes. Nat. Biotechnol. *27*, 59–65.

31. Van den Broeck, W., Derore, A., and Simoens, P. (2006). Anatomy and nomenclature of murine lymph nodes: descriptive study and nomenclatory standardization in BALB/cAnNCrl mice. J. Immunol. Methods *312*, 12–19.

32. Lai, Z., Yin, H., Cabrera-Pérez, J., Guimaro, M.C., Afione, S., Michael, D.G., Glenton, P., Patel, A., Swaim, W.D., Zheng, C., et al. (2016). Aquaporin gene therapy corrects Sjögren's syndrome phenotype in mice. Proc. Natl. Acad. Sci. USA *113*, 5694–5699.

33. Nguyen, C.Q., Yin, H., Lee, B.H., Chiorini, J.A., and Peck, A.B. (2011). IL17: potential therapeutic target in Sjögren's syndrome using adenovirus-mediated gene transfer. Lab. Invest. *91*, 54–62.

34. Roescher, N., Vosters, J.L., Alsaleh, G., Dreyfus, P., Jacques, S., Chiocchia, G., Sibilia, J., Tak, P.P., Chiorini, J.A., Mariette, X., and Gottenberg, J.-E. (2014). Targeting the splicing of mRNA in autoimmune diseases: BAFF inhibition in Sjögren's syndrome as a proof of concept. Mol. Ther. *22*, 821–827.

35. Roescher, N., Vosters, J.L., Yin, H., Illei, G.G., Tak, P.P., and Chiorini, J.A. (2011). Effect of soluble ICAM-1 on a Sjögren's syndrome-like phenotype in NOD mice is disease stage dependent. PLoS ONE *6*, e19962.

36. Vosters, J.L., Roescher, N., Illei, G.G., Chiorini, J.A., and Tak, P.P. (2012). TACI-Fc gene therapy improves autoimmune sialadenitis but not salivary gland function in non-obese diabetic mice. Oral Dis. *18*, 365–374.

37. Yin, H., Nguyen, C.Q., Samuni, Y., Uede, T., Peck, A.B., and Chiorini, J.A. (2012). Local delivery of AAV2-CTLA4IgG decreases sialadenitis and improves gland function in the C57BL/6.NOD-Aec1Aec2 mouse model of Sjogren's syndrome. Arthritis Res. Ther. *14*, R40.

38. Adriaansen, J., Perez, P., Zheng, C., Collins, M.T., and Baum, B.J. (2011). Human parathyroid hormone is secreted primarily into the bloodstream after rat parotid gland gene transfer. Hum. Gene Ther. *22*, 84–92.

39. Liu, X., Yan, Z., Luo, M., and Engelhardt, J.F. (2006). Species-specific differences in mouse and human airway epithelial biology of recombinant adeno-associated virus transduction. Am. J. Respir. Cell Mol. Biol. *34*, 56–64.

OMTM, Volume *19*

Supplemental Information

Transduction of Salivary Gland Acinar Cells with a Novel AAV Vector 44.9

Giovanni Di Pasquale, Paola Perez Riveros, Muhibullah Tora, Tayyab Sheikh, Aran Son, Leyla Teos, Brigitte Grewe, William D. Swaim, Sandra Afione, Changyu Zheng, Shyh-Ing Jang, Akiko Shitara, Ilias Alevizos, Roberto Weigert, and John A. Chiorini

Figure S1. Acinar or ductal salivary glands cells have distinct membrane distribution of fluorescent reporter

Salivary glands from a mouse expressing the mTomato reporter (red) top right image, were fixed, processed for indirect immunofluorescence, labeled with the acinar marker NKCC1 (green), top left image, and phalloidin (blue), lower left image, then imaged by confocal microscopy, as described in the M&M section. In the merge image, lower right, NKCC1 positive acini (dashed white line) can be distinguished by their distribution of phalloidin from ducts (dashed yellow line) by their characteristic morphology and lumen structure. Yellow and white arrows in the insets highlight the basal membrane of the duct and acinar cell, respectively. Ductal cells exhibit a characteristic thicker distribution of the mTomato reporter membrane reporter due to the folding of the plasma membrane as also seen with phalloidin. Scale bar 10 µm.

Figure S2. Images of the salivary glands in mTomato/mGFP mice. The plasma membrane of salivary glands of CRE tomato/GFP mice has a strong red fluorescence that becomes green upon introduction of the CRE recombinase. Acini and ducts are structurally distinct as highlighted in the blue and yellow circles respectively (top image). Magnified images from Fig. 1 of positive acinar cells in mice transduced with the indicated AAV vector (lower images).

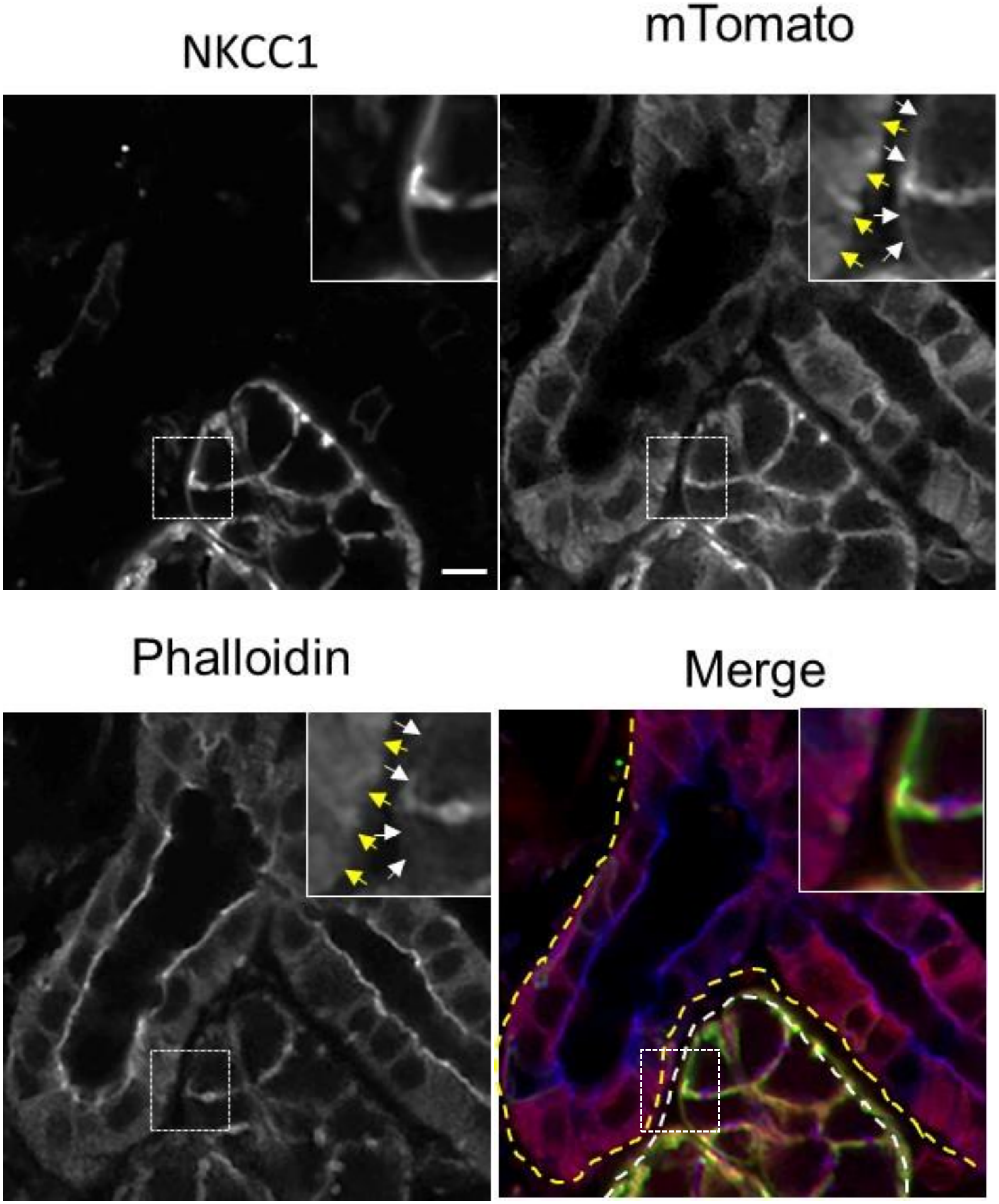

Supplemental Figure 1

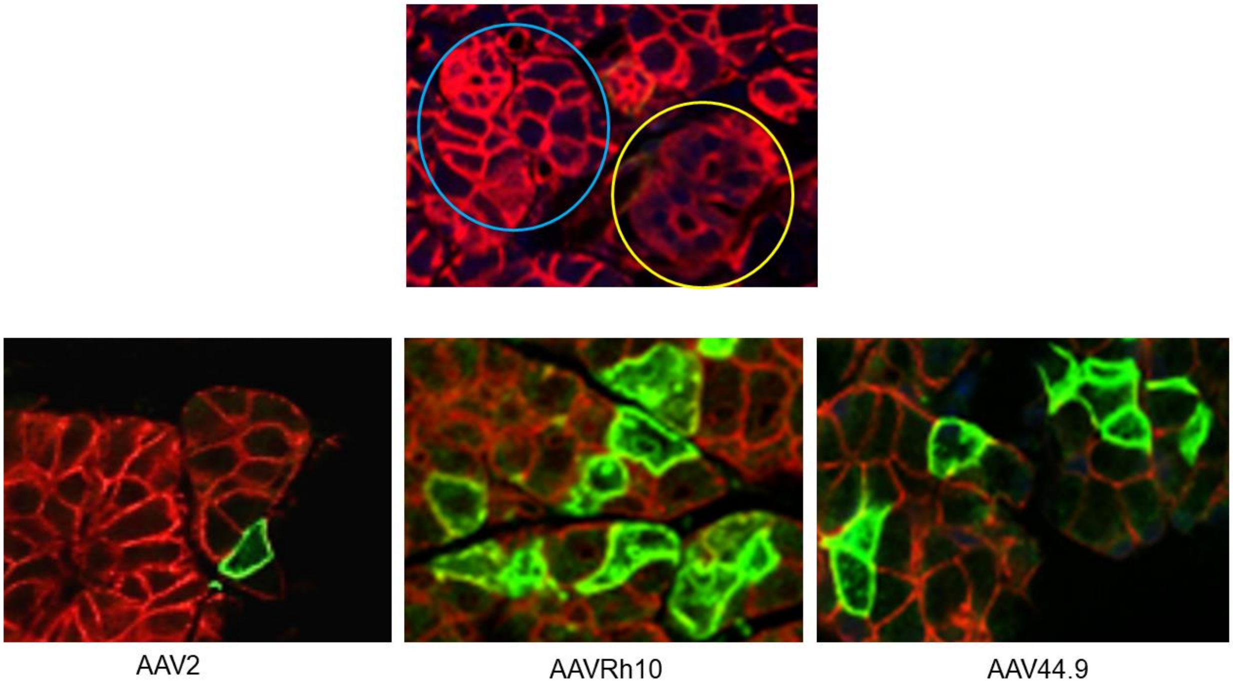

Supplemental Figure 2
